# Effectiveness of an intervention in increasing the provision of preventive care by community mental health services: a non-randomized, multiple baseline implementation trial

**DOI:** 10.1186/s13012-016-0408-4

**Published:** 2016-04-02

**Authors:** Kate M. Bartlem, Jenny Bowman, Megan Freund, Paula M. Wye, Daniel Barker, Kathleen M. McElwaine, Luke Wolfenden, Elizabeth M. Campbell, Patrick McElduff, Karen Gillham, John Wiggers

**Affiliations:** 1 School of Psychology, Faculty of Science and Information Technology, The University of Newcastle, University Drive, Callaghan, NSW 2308 Australia; 2 Population Health, Hunter New England Local Health District, Booth Building, Wallsend Health Services, Longworth Avenue, Wallsend, NSW 2287 Australia; 3 Hunter Medical Research Institute, Clinical Research Centre, Level 3 John Hunter Hospital, Lookout Road, New Lambton Heights, NSW 2305 Australia; 4 School of Medicine and Public Health, Faculty of Health, The University of Newcastle, University Drive, Callaghan, NSW 2308 Australia

## Abstract

**Background:**

Relative to the general population, people with a mental illness are more likely to have modifiable chronic disease health risk behaviours. Care to reduce such risks is not routinely provided by community mental health clinicians. This study aimed to determine the effectiveness of an intervention in increasing the provision of preventive care by such clinicians addressing four chronic disease risk behaviours.

**Methods:**

A multiple baseline trial was undertaken in two groups of community mental health services in New South Wales, Australia (2011–2014). A 12-month practice change intervention was sequentially implemented in each group. Outcome data were collected continuously via telephone interviews with a random sample of clients over a 3-year period, from 6 months pre-intervention in the first group, to 6 months post intervention in the second group. Outcomes were client-reported receipt of assessment, advice and referral for tobacco smoking, harmful alcohol consumption, inadequate fruit and/or vegetable consumption and inadequate physical activity and for the four behaviours combined. Logistic regression analyses examined change in client-reported receipt of care.

**Results:**

There was an increase in assessment for all risks combined following the intervention (18 to 29 %; OR 3.55*, p* = 0.002: *n* = 805 at baseline, 982 at follow-up). No significant change in assessment, advice or referral for each individual risk was found.

**Conclusions:**

The intervention had a limited effect on increasing the provision of preventive care. Further research is required to determine how to increase the provision of preventive care in community mental health services.

**Trial registration:**

Australian and New Zealand Clinical Trials Registry ACTRN12613000693729

## Background

People with a mental illness experience a disproportionately higher chronic disease burden when compared to the general population and a substantially reduced life expectancy as a consequence [1–7]. Such poor health outcomes are contributed to by a higher prevalence of modifiable chronic disease health risk behaviours, including smoking [8–13], inadequate nutrition [13–16], harmful alcohol consumption [8, 13, 14, 17, 18] and inadequate physical activity [9, 13, 17, 19, 20].

Routine care delivery by clinicians to address chronic disease risk behaviours (preventive care) is recommended for all health services [21–27], including mental health services [28–39]. Such care is recommended to involve, at a minimum, clinician assessment of client risk status and, for clients identified as being at risk, provision of advice and referral to specialist preventive care services [40, 41]. Although community mental health services represent a key setting for the provision of preventive care [42], the provision of such care in this setting is both variable and sub-optimal [42–47].

Cochrane systematic review evidence supports the efficacy of a range of strategies in improving the provision of recommended elements of clinical care, with such strategies including leadership and consensus [48], enablement of systems and procedures [49–51], training and education [52], monitoring and feedback [53, 54], provision of practice change resources such as educational materials and clinical practice guidelines [55] and practice change support such as educational outreach or academic detailing [56]. In intervention trials in general health services, implementation of such strategies has been associated with increases in care delivery for smoking [57–62], at-risk alcohol consumption [63] and multiple health risk behaviours [64].

Only one study could be identified that assessed the effectiveness of a practice change intervention in increasing the provision of preventive care for multiple health risks in a community mental health setting [65]. A single group pre-post study was undertaken in two USA services of a 6-month intervention to increase the provision of risk assessment regarding a number of cardiovascular disease risks (tobacco smoking and non-behavioural risks e.g. blood pressure and cholesterol) and the sending of a letter to clients’ primary care providers. The intervention practice change strategies included staff education, an electronic screening tool and a template for a standard communication letter. A random sample of clients’ medical records was audited before (*n* = 129) and after (*n* = 117) the intervention. The proportion of clients screened for smoking by psychiatrists, mental health nurses and case managers increased from 76 to 89 %, while the proportion of clients for whom a letter was sent to their primary care provider increased from 19 to 32 % [65].

Further research is needed to examine whether a practice change intervention can improve the provision of a broader range of preventive care elements for the most common chronic disease risk behaviours. To address this need, a study was undertaken to determine the effectiveness of a multi-strategic practice change intervention in increasing the provision of three elements of preventive care (risk assessment, brief advice and referral) by community mental health clinicians for four health risk behaviours (smoking, inadequate fruit and vegetable consumption, harmful alcohol consumption and inadequate physical activity).

## Methods

### Study design and setting

A multiple baseline trial [66] was undertaken involving a 12-month intervention delivered sequentially in two groups of community-based mental health services. Outcome data were collected for both groups from 6 months prior to the implementation of the intervention in the first group of services, and continued until 6 months after the completion of the intervention in the second group of services (36-month study period). Further details of the study design and methods have been reported previously [67]. The study was undertaken in a single regional health district in New South Wales, Australia. Ethics approval was obtained from the Hunter New England Human Research Ethics Committee (approval no. 09/06/17/4.03) and the University of Newcastle Human Research Ethics Committee (approval no. H-2010-1116). The trial was registered with the Australian and New Zealand Clinical Trials Registry (ACTRN12613000693729).

### Participants

#### Community mental health services

All community mental health services (*n* = 19) in the health district that provided ambulatory care to clients 18 years of age or greater, and were not involved in a pilot of this study, were included and allocated to two service groups (*n* = 7; *n* = 12) based on their geographic location and associated administrative boundaries. The services provided general adult community mental health care, and care for specific client populations, including older persons, psychiatric rehabilitation, early diagnosis, comorbid substance use, eating disorders and borderline personality disorder.

#### Clinicians

All clinicians and managers in the eligible services (psychiatrists, psychologists, social workers, dietitians, nurses, occupational therapists and health service managers) received the intervention. The services were staffed with approximately 220 clinicians, predominantly nurses (40 %), psychiatrists (15 %) and psychologists (15 %).

#### Clients

All clients who attended a face-to-face individual clinical appointment were eligible to receive preventive care.

Clients were eligible to be selected for data collection if they were 18 years or older, had attended at least one face-to-face individual appointment with an eligible service within the previous 2 weeks, had not previously been selected to participate in the study and had not been identified by their clinician as too unwell to participate. Of such clients, additional eligibility criteria were as follows: English speaking, not living in aged care facilities or gaol and being physically and mentally capable of responding to the survey items.

### Intervention

#### Preventive care

Clinicians were asked to routinely provide preventive care based on the recommended ‘2As and R’ model, a model that includes three elements of care [40, 68, 69]:

##### Assessment

Assessment of client risk status for each of the four health risk behaviours based on levels of risk defined in Australian national guidelines [70–73]

##### Brief advice

Provision of advice to clients assessed as being at-risk to modify their risk to comply with the Australian national guidelines [70–73] and the benefits of doing so

##### Referral

Offer of a referral for clients with risks to evidence-based state-wide telephone support services for smoking (New South Wales [NSW] Quitline) and for physical inactivity and inadequate nutrition (NSW Get Healthy Service). For all risk behaviours, referral could additionally be provided to the client’s primary care provider (general practitioner or Aboriginal medical service) or local referral options (e.g. dietitians, exercise groups and drug and alcohol services)

#### Practice change intervention

The following multi-strategic clinical practice change intervention, informed by research and reviews of the clinical practice change literature [48, 52, 53, 55, 74], was implemented:

1. Leadership and consensus
  A district-wide policy and key performance indicators regarding the provision of preventive care were implemented based on consultation with health district executives, senior clinicians and managers.
2. Enabling systems and procedures
  A tool was incorporated into the electronic medical record used by all clinicians to enable standardized assessment and recording of risk status and subsequent provision of preventive care; the automated production of a tailored client risk reduction information sheet and referral letter to the clients’ primary care provider; and prompts to deliver brief advice and referral where clients were identified as at-risk.
3. Clinician and manager training
  Clinicians and managers were provided online educational competency-based training of approximately 2-h duration, addressing the following: the provision of preventive care, including the ‘2As and R’ model; policy guidelines and performance indicators; and the recording of such care in the standardized electronic tool. Managers were additionally provided with a 2-h, face-to-face training session regarding care delivery performance monitoring and feedback and leadership in preventive care.
4. Monitoring and feedback
  Modifications were made to the electronic medical record to allow automated production of monthly performance reports regarding the provision of preventive care at the service level. Reports were provided to and discussed with managers monthly.
5. Provision of practice change resources
  An e-mail helpline and internet resource site were established, and monthly newsletters and tip-sheets and a resource pack including a process flowchart, a guide, information on each risk behaviour, fax-based referral forms for telephone referral services, and a paper-based preventive care assessment tool for use during home visits were distributed to clinicians and managers.
6. Practice change support
  Project personnel (approximately one full time equivalent per group) were allocated to support intervention delivery, including monthly face-to-face visits with managers and clinicians, and fortnightly support phone calls and/or e-mails to managers. The project personnel discussed the feedback reports and provided both proactive and reactive support to managers and clinicians.

### Data collection procedures

#### Recruitment

Each week, a random sample of 40 eligible adult clients (20 from each of the two groups; approximately 7 % of eligible clients per week) was drawn from the health service electronic medical records. These clients were mailed an information statement and contacted by telephone by trained interviewers, blind to group allocation, to confirm eligibility.

Eligible clients were asked to participate in a telephone interview regarding their health behaviour risk status, the preventive care they had received for such risks and a number of demographic and clinical characteristics. The interview was approximately 20 min in length.

#### Measures

##### Client characteristics

Clients reported their Aboriginal and/or Torres Strait Islander status, highest education level attained, employment status, marital status and physical or psychiatric conditions for which they had received health care within the previous 2 months. Client age, gender, postcode, and the number of community mental health appointments within the last 12 months were obtained from the electronic medical record.

##### Client health behaviour risk status

Clients reported their health behaviour risk status for the month prior to seeing their community mental health clinician. Survey items were based on recommended assessment tools [75–78] and previous community surveys [13, 46, 47, 79]. In line with national guidelines, clients were defined as being at-risk if they reported smoking any tobacco products [70], consuming less than two serves of fruit or five serves of vegetables per day [72], consuming more than two standard drinks on average per day or four or more standard drinks on any one occasion [71] or engaging in less than 30 min of physical activity on at least 5 days of the week [73].

##### Client-reported provision of preventive care

*Assessment*. Clients were asked to report whether, during a community mental health appointment, a clinician had asked about their smoking status, fruit and vegetable intake, alcohol consumption and physical activity (yes, no, don’t know for each).

*Brief advice*. Clients classified as being at-risk for a health risk behaviour(s) based on their self-report were asked whether their community mental health clinician had advised them to modify their behaviour(s) (yes, no, don’t know for each).

*Referral*. Clients classified as having at least one risk were asked whether their community mental health clinician had offered to send their primary care provider a letter summarizing their health behaviour risks and the preventive care provided. Clients classified as at risk for a health risk behaviour(s) were also asked whether their clinician had provided each of the following forms of referral (‘yes, no, or don’t know’):

- Spoke about the NSW Quitline telephone support service (for smoking); or the NSW Get Healthy Service (for clients with inadequate fruit and vegetable intake or inadequate physical activity);
- Offered to arrange for a telephone support service (NSW Quitline or NSW Get Healthy Service) to call them;
- Recommended speaking to their primary care provider about their health risk behaviour(s); and
- Advised to use any other supports to make changes to their health behaviour(s) (e.g. dietitian, physical activity classes, website).

##### Intervention delivery

Project personnel recorded the implementation of each practice change strategy for each service on a monthly basis.

### Statistical analysis

Analyses were undertaken using SAS V9.4. Residential postcode was used to classify client residential geographic location [80] and socio-economic status [81]. Chi square tests were used to compare consenters and non-consenters regarding age group, gender, remoteness, disadvantage and number of appointments*.* Descriptive statistics were used to describe participating client characteristics, health behaviour risk status and receipt of preventive care. For care receipt items, clients who responded ‘don’t know’ were classified as not having received care. For each of the four behaviours, referral items were combined to create a single variable reflecting receipt of any form of referral.

A variable was created to reflect client receipt of assessment for all four risk behaviours. Separate variables were also created to reflect client receipt of brief advice for all behaviours for which they were at risk and receipt of any referral for all behaviours for which they were at risk (‘all risks combined’).

#### Intervention effectiveness

Logistic regression models were used to examine changes in the prevalence of preventive care delivery between the baseline and follow-up periods for the two service groups combined and for each of the two service groups individually. Separate models were developed to examine change in delivery of each of the three elements of preventive care for each of the four risk behaviours and for all four behaviours combined; and for the delivery of a letter to the client’s primary care provider (16 models in total). Five models were developed for each of the assessment and brief advice outcomes, and 6 models were developed for the referral outcome. For all models, intervention effect was defined as the difference in prevalence of preventive care delivery from the baseline to the post-intervention periods, adjusted for service group, time and the number of client visits to the service in the prior 12 months (the latter added to account for any introduced selection bias). Analyses are reported using data collected during the baseline and follow-up periods. While all models were also analysed incorporating the intervention period data, as the results did not differ, the simpler method is presented. A significance level of *α* = 0.01 was used to adjust for multiple testing [82]. As simple random sampling of community mental health clients was used (see “Recruitment” section), there was no need to adjust for clinician, community mental health service or any other natural clustering that occurs within the community. An unadjusted analysis provides an unbiased estimate of the statistics of interest.

## Results

### Sample characteristics

Of the 3764 clients selected to participate, 2817 were able to be contacted by telephone (75 %), and 375 were identified as ineligible upon contact. Of the 2442 eligible potential participants, 1787 (73 %) consented to participate and completed the survey (*n* = 805 at baseline, *n* = 982 at follow-up). There were no significant differences in the characteristics between consenting and non-consenting clients. Characteristics of the sample are presented in Table 1.

**Table 1:** Sample characteristics by group and time

		Group 1		Group 2	
Variable	Class	Baseline (*n* = 110)	Follow-up (*n* = 677)	Baseline (*n* = 695)	Follow-up (*n* = 305)
Gender	Male	49 (45 %)	310 (46 %)	327 (47 %)	143 (47 %)
Age	<40	53 (48 %)	320 (47 %)	366 (53 %)	160 (52 %)
	40–49	19 (17 %)	154 (23 %)	151 (22 %)	61 (20 %)
	50–59	22 (20 %)	109 (16 %)	94 (14 %)	46 (15 %)
	60+	16 (15 %)	94 (14 %)	84 (12 %)	38 (12 %)
Index of disadvantage^a^	Lower half	92 (84 %)	606 (90 %)	363 (53 %)	154 (51 %)
	Higher half	17 (16 %)	69 (10 %)	328 (47 %)	150 (49 %)
Remoteness^b^	Major cities	0 (0 %)	5 (0.7 %)	526 (76 %)	197 (65 %)
	Regional/remote	109 (100 %)	670 (99 %)	165 (24 %)	107 (35 %)
Aboriginality	Aboriginal and/or Torres Strait Islander	8 (7.3 %)	88 (13 %)	35 (5.0 %)	21 (6.9 %)
Marital status	Not living with a partner	69 (63 %)	426 (63 %)	530 (76 %)	213 (70 %)
	Living with partner	41 (37 %)	248 (37 %)	165 (24 %)	92 (30 %)
Education	Some high school or less	66 (60 %)	338 (50 %)	319 (46 %)	129 (42 %)
	Completed high school	14 (13 %)	93 (14 %)	134 (19 %)	49 (16 %)
	TAFE certificate or diploma	19 (17 %)	171 (25 %)	157 (23 %)	88 (29 %)
	University, CAE, degree or higher	11 (10 %)	75 (11 %)	84 (12 %)	39 (13 %)
Employment	Employed	17 (15 %)	191 (28 %)	153 (22 %)	79 (26 %)
	Not working	64 (58 %)	307 (45 %)	381 (55 %)	151 (50 %)
	Retired	11 (10 %)	68 (10 %)	58 (8.3 %)	27 (8.9 %)
	Other	18 (16 %)	111 (16 %)	103 (15 %)	48 (16 %)
Psychiatric diagnosis^c^	Depression	54 (49 %)	443 (65 %)	392 (56 %)	198 (65 %)
	Bipolar disorder	24 (22 %)	77 (11 %)	139 (20 %)	70 (23 %)
	Schizophrenia/psychosis	17 (15 %)	82 (12 %)	207 (30 %)	54 (18 %)
	Anxiety	29 (26 %)	268 (40 %)	226 (33 %)	130 (43 %)
Appointments in previous 12 months	1–2	34 (31 %)	532 (79 %)	159 (23 %)	152 (50 %)
	3–11	47 (43 %)	140 (21 %)	221 (32 %)	109 (36 %)
	12+	29 (26 %)	5 (<1 %)	315 (45 %)	44 (14 %)
Risk status	Smoking	49 (45 %)	340 (50 %)	355 (51 %)	122 (40 %)
	Physical inactivity	49 (45 %)	232 (34 %)	332 (48 %)	134 (44 %)
	Alcohol over consumption	50 (45 %)	294 (43 %)	309 (44 %)	126 (41 %)
	Fruit and vegetable under consumption	98 (89 %)	557 (82 %)	611 (88 %)	251 (82 %)
Number of risks	0	4 (3.6 %)	43 (6.4 %)	26 (3.7 %)	19 (6.2 %)
	1	22 (20 %)	153 (23 %)	117 (17 %)	67 (22 %)
	2	35 (32 %)	235 (35 %)	241 (35 %)	111 (36 %)
	3	35 (32 %)	184 (27 %)	236 (34 %)	88 (29 %)
	4	14 (13 %)	62 (9.2 %)	75 (11 %)	20 (6.6 %)

Denominator varies by item due to non-responses

^a^SEIFA index of disadvantage: lower NSW half (≤991); higher NSW half (>991)

^b^Accessibility/Remoteness Index of Australia (ARIA)

^c^Percentages do not add to 100 % as participants could elect more than one diagnosis. A number of participants reported no psychiatric diagnoses (group 1: 7 at baseline, 53 at follow-up; group 2: 52 at baseline, 21 at follow-up)

### Intervention effectiveness

For both groups combined, there was a significant increase in the prevalence of one of the 16 outcome measures. From baseline to follow-up, there was an increase in assessment for all risks combined (18 to 29 %; OR 3.55*, p* = 0.002) (Table 2).

**Table 2:** Levels of preventive care at baseline and follow-up, and estimates of the intervention effect, for both groups combined

Outcome	Baseline (*n* = 805)	Follow-up (*n* = 982)	Intervention effect	*p* value
			Odds ratio (95 % CI)	
Risk assessment				
Smoking	610 (76 %)	805 (82 %)	0.87 (0.41–1.83)	0.712
Nutrition	191 (24 %)	357 (36 %)	2.62 (1.25–5.52)	0.011
Alcohol	632 (79 %)	820 (84 %)	1.83 (0.85–3.96)	0.123
Physical activity	467 (58 %)	575 (59 %)	1.03 (0.54–1.95)	0.934
All risks combined	146 (18 %)	297 (30 %)	3.55 (1.56–8.08)	0.002
Brief advice^a^				
Smoking	275 (67 %)	298 (65 %)	1.89 (0.73–4.92)	0.190
Nutrition	186 (26 %)	267 (33 %)	2.43 (1.11–5.33)	0.026
Alcohol	222 (62 %)	238 (57 %)	1.44 (0.54–3.81)	0.468
Physical activity	234 (61 %)	191 (52 %)	0.38 (0.14–0.99)	0.048
All applicable risks combined	185 (24 %)	250 (27 %)	1.33 (0.62–2.87)	0.468
Referral^a^				
Smoking referral (any)^b^	173 (42 %)	224 (48 %)	2.16 (0.86–5.4)	0.101
Nutrition referral (any)^c^	128 (18 %)	174 (22 %)	1.36 (0.56–3.29)	0.493
Alcohol referral (any)^d^	127 (35 %)	153 (36 %)	1.01 (0.37–2.75)	0.981
Physical activity referral (any)^c^	123 (32 %)	113 (31 %)	1.04 (0.38–2.84)	0.947
Referral—all applicable risks (any)^b,c,d^	0 (0 %)	12 (1.3 %)	0.93 (0.19–4.5)	0.925
Letter to primary care provider	206 (26 %)	227 (23 %)	0.66 (0.32–1.34)	0.249
Additional referral outcomes^a,e^				
Smoking arrange^f^	11 (2.7 %)	11 (2.4 %)		
Nutrition arrange^f^	5 (0.7 %)	40 (5.0 %)		
Physical activity arrange^f^	7 (1.8 %)	11 (3.0 %)		
Smoking—primary care provider	52 (13 %)	79 (17 %)		
Nutrition—primary care provider	6 (0.8 %)	25 (3.1 %)		
Alcohol—primary care provider	38 (11 %)	41 (9.8 %)		
Physical activity—primary care provider	5 (1.3 %)	6 (1.6 %)		

^a^Of participants who reported being at-risk for each relevant behaviour

^b^Includes the following: clinician spoke about NSW Quitline, offered to arrange for NSW Quitline to call them, recommended they speak to their primary care provider or advised them to use any other support

^c^Includes the following: clinician spoke about NSW Get Healthy Service, offered to arrange for NSW Get Healthy Service to call them, recommended they speak to their primary care provider or advised them to use any other support

^d^Includes the following: recommended they speak to their primary care provider or advised them to use any other support

^e^Intervention effect could not be modelled meaningfully due to small sample size

^f^Includes the following: clinician offered to arrange for NSW Quitline to call them (smoking) or for NSW Get Healthy Service to call them (nutrition and/or physical activity)

When examined separately for each of the two service groups, there was an increase in the prevalence of one outcome for group 1. From baseline to follow-up, there was an increase in the assessment of nutrition (18 to 32 %; OR 5.55, *p* = 0.001). No increases in care were identified for group 2 individually (Table 3).

**Table 3:** Levels of preventive care at baseline and follow-up, and estimates of the intervention effect, for each group

	Group 1				Group 2			
Outcome	Baseline (*n* = 110)	Follow-up (*n* = 677)	Intervention effect	*p* value	Baseline (*n* = 695)	Follow-up (*n* = 303)	Intervention effect	*p* value
			Odds ratio (95 % CI)				Odds ratio (95 % CI)	
Risk assessment								
Smoking	95 (86 %)	554 (82 %)	0.61 (0.22–1.68)	0.340	515 (74 %)	251 (82 %)	1.27 (0.50–3.23)	0.621
Nutrition	17 (15 %)	241 (36 %)	2.83 (1.11–7.22)	0.030	174 (25 %)	116 (38 %)	2.47 (0.98–6.19)	0.054
Alcohol	89 (81 %)	554 (82 %)	0.78 (0.30–2.04)	0.613	543 (78 %)	266 (87 %)	2.78 (1.02–7.56)	0.045
Physical activity	66 (60 %)	387 (57 %)	0.72 (0.33–1.57)	0.410	401 (58 %)	188 (62 %)	1.49 (0.65–3.39)	0.343
All risks combined	14 (13 %)	202 (30 %)	2.65 (0.99–7.08)	0.053	132 (19 %)	95 (31 %)	3.75 (1.36–10.34)	0.011
Brief advice^a^								
Smoking	34 (61 %)	217 (64 %)	1.46 (0.47–4.52)	0.510	241 (68 %)	81 (66 %)	1.93 (0.55–6.80)	0.308
Nutrition	18 (18 %)	177 (32 %)	5.55 (1.98–15.55)	0.001	168 (27 %)	90 (36 %)	2.07 (0.78–5.52)	0.144
Alcohol	26 (52 %)	158 (54 %)	1.51 (0.50–4.55)	0.466	196 (63 %)	80 (63 %)	1.26 (0.35–4.56)	0.726
Physical activity	28 (57 %)	120 (52 %)	0.42 (0.12–1.49)	0.179	206 (62 %)	71 (53 %)	0.38 (0.11–1.31)	0.125
All applicable risks combined	15 (14 %)	162 (26 %)	2.32 (0.85–6.33)	0.100	170 (25 %)	88 (31 %)	1.08 (0.42–2.80)	0.876
Referral^a^								
Smoking referral (any)^b^	21 (38 %)	160 (47 %)	1.49 (0.49–4.56)	0.482	152 (43 %)	64 (52 %)	2.64 (0.80–8.70)	0.110
Nutrition referral (any)^c^	11 (11 %)	114 (20 %)	1.59 (0.54–4.74)	0.402	117 (19 %)	60 (24 %)	1.32 (0.44–3.97)	0.618
Alcohol referral (any)^d^	19 (38 %)	102 (35 %)	0.84 (0.27–2.68)	0.773	108 (35 %)	51 (40 %)	1.50 (0.40–5.57)	0.544
Physical activity Referral (any)^c^	9 (18 %)	68 (29 %)	6.37 (1.44–28.25)	0.015	114 (34 %)	45 (34 %)	0.54 (0.16–1.88)	0.334
Referral—all applicable risks (any)^b,c,d^	0 (0 %)	8 (1.3 %)	1.02 (0.02–58.88)	0.993	0 (0 %)	4 (1.4 %)	-^e^	-^e^
Letter to primary care provider	44 (40 %)	156 (23 %)	0.81 (0.35 –1.91)	0.637	162 (23 %)	71 (23 %)	1.04 (0.40–2.68)	0.940

^a^Of participants who reported being at-risk for each relevant behaviour

^b^Includes the following: clinician spoke about NSW Quitline, offered to arrange for NSW Quitline to call them, recommended they speak to their primary care provider or advised them to use any other support

^c^Includes the following: clinician spoke about NSW Get Healthy Service, offered to arrange for NSW Get Healthy Service to call them, recommended they speak to their primary care provider or advised them to use any other support

^d^Includes the following: recommended they speak to their primary care provider or advised them to use any other support

^e^Intervention effect could not be modelled meaningfully due to small sample size

### Intervention implementation

The implementation of intervention strategies is shown in Table 4. Overall, the intervention strategies were not delivered as intended. On average per month for the two service groups combined, the proportion of services, managers or clinicians that received each strategy ranged from 63 % (performance reported discussed with managers) to 78 % (fortnightly phone/email support). Group 1 received fewer monthly intervention strategies on average. The proportion of services, managers or clinicians that received each strategy in group 1 ranged from 33 % (performance reports discussed with managers) to 69 % (face-to-face visits with clinicians), compared to 72 % (performance reports discussed with managers) to 83 % (fortnightly phone or email support for managers) in group 2 (Table 4).

**Table 4:** Summary of intervention strategy implementation

Monthly intervention strategies	Average number who received strategy per month^a^		
	Group 1^b^	Group 2^c^	Overall
Practice change support officer contacts			
Face-to-face visits (managers)	1.6/3 (53 %)	7.6/10 (76 %)	9.2/13 (71 %)
Face-to-face visits (clinicians)^d^	4.8/7 (69 %)	8.7/12 (73 %)	13.4/19 (71 %)
Fortnightly phone/email support (managers)	1.8/3 (60 %)	8.3/10 (83 %)	10.1/13 (78 %)
Monitoring and feedback			
Performance reports provided (managers)^e^	1.4/3 (47 %)	7.5/10 (75 %)	8.9/13 (68 %)
Performance reports discussed with (managers)^e^	1.0/3 (33 %)	7.2/10 (72 %)	8.2/13 (63 %)
Practice change resources			
Tips and updates sheets provided to clinicians (service)^f^	3.4/7 (49 %)	9.0/12 (75 %)	12.4/19 (65 %)
Newsletter provided to clinicians (service)^f^	4.4/7 (63 %)	9.0/12 (75 %)	13.4/19 (71 %)
One-off intervention strategies^g^	Month by which majority of target (80 % ) received strategy^h^		
	Group 1^b^	Group 2^c^	Overall
Clinician and manager training			
Manager training (managers)	5/12	4/12	5/12
Online training (managers)	n/a^i^	4/12	5/12
Online training (clinicians)	7/12	5/12	6/12
Practice change resources			
Provision of resource pack (service)^f^	4/12	1/12	3/12

^a^Average number of targets of the intervention strategy (services or managers) who received each strategy per month

^b^Includes 7 services with a total of 3 managers and 52 clinicians

^c^Includes 12 services, with a total of 10 managers and 165 clinicians

^d^Recorded at service level as support officer made available to all clinicians at relevant service

^e^Due to complications with the software used for performance monitoring and feedback, this strategy was not available for 6/12 months of the intervention period in group 1 and 3/12 months in group 2

^f^Recorded at service level as resource provided to the service to distribute to individual clinicians

^g^The following strategies were implemented across the health district prior to intervention implementation: district wide preventive care policy, key performance indicators (based on consultation with health district executives, senior clinicians and managers), tool incorporated into the electronic medical record, e-mail helpline and an internet resource site

^h^Intervention month in which the majority of services, managers or clinicians (80 %) had received each ‘one-off’ intervention strategy

^i^<80 % of managers in group 1 completed the online training modules by the completion of the intervention. By month 5, 2/3 managers had completed the modules. The third manager did not complete the modules by the completion of the intervention

Group 2 generally received one-off strategies (training and practice change resources) at an earlier stage during the intervention. For instance, the majority (80 % or more) of services in group 2 had received the resource pack by the end of month 1, compared to the end of month 4 in group 1 (Table 4).

## Discussion

This is the first study to examine the effectiveness of a multi-strategy practice change intervention in increasing the provision of multiple elements of preventive care for multiple chronic disease health risk behaviours within a community mental health care setting. Overall, the study had a limited effect in increasing the provision of elements of care, with an effect observed only for the assessment of risk status for all behaviours combined. Further research is required to identify strategies for improving the delivery of chronic disease preventive care in these settings.

One previous study has examined the effectiveness of similar practice change strategies in increasing the delivery of cardiovascular disease risk screening in community mental health services [65]. The single group pre-post study conducted in the USA reported an increase in assessment of smoking status (13 %), and for providing a letter to the clients’ primary care provider (13 %). In comparison, in our controlled trial, we found an effect for assessment across risks, but not smoking, and not for providing a letter to the primary care provider. The absence of a control group in the previous study precludes a direct comparison of effect between the two studies.

The intervention in the current study involved the use of practice change strategies previously found to be effective in general health care services but not trialled in mental health services [57–64]. Importantly, the same intervention strategies were implemented in a contemporaneous study conducted in general community health services (addressing physical health care) within the same health district in which the current study was conducted [64]. That study found, using the same outcome measures and intervention approach, increases in care provision for six out of ten assessment and advice measures of preventive care (assessment of fruit and vegetable consumption, physical activity and for all risks; and brief advice for inadequate fruit and vegetable consumption, harmful alcohol consumption and for all risks [64]). However, consistent with this trial, no effect was found for provision of any element of smoking care or of referral.

The need to address the clinical, professional, cultural and organizational factors [83–85] that distinguish community mental health service delivery from the delivery of general community health services may have contributed to the contrasting findings. The findings suggest that a greater understanding of the context and barriers to the provision of preventive care in community mental health services is required. Similarly, tailoring of recommendations regarding the provision of care addressing chronic disease risk behaviours that can be operationalized in the context and circumstances of community mental health services also appears warranted, as does tailoring of the practice change strategies to support the delivery of such care. The use of systematic and theory-based methods for identifying barriers and designing interventions, such as the Theoretical Domains Framework [86], may provide a useful approach to achieving this.

No increases in either brief advice or referral were identified for any of the four health risk behaviours. Such findings are of significance as any benefit in terms of reduction in risk of chronic disease is dependent upon either or both of these elements of care [40, 41, 87]. Both elements of care have been shown to be effective in reducing the prevalence of health risks for clients of general health services [88–96]. Previous research has identified a number of barriers to mental health clinician provision of risk advice, including clinician attitudes regarding their role in providing preventive care [97–99] and a lack of training in how to provide preventive care [42, 97]. Previous research has also identified a lack of referral options as a barrier to mental health clinicians providing referrals [47, 100]. The current study sought to address barriers to both elements of preventive care through a comprehensive suite of practice change strategies including a policy, electronic prompts, fax referral forms to free public evidence-based specialist risk reduction services, automated production of referral letters to primary care providers, clinician training and education, monthly performance monitoring feedback reports and allocated practice change support personnel for 12 months. Notwithstanding the comprehensiveness of these strategies, they may not have been of a sufficient dose (e.g. frequency of contact with allocated practice change support) or of sufficient length.

Additional factors also may have impeded the clinicians’ ability to refer clients. In USA primary care services, additional strategies have been found to be effective in increasing referrals to tobacco quitlines and community behavioural counselling services including the use of financial incentives [101], and automatic, electronic referral processes [102]. However, the effectiveness of these strategies in increasing referrals regarding chronic disease risk behaviours is yet to be examined in community mental health services.

The study outcomes should be interpreted in light of a number of its methodological characteristics. First, although the study was conducted across a number of community mental health services in urban, regional and rural locations, all the services were located within one health district, potentially limiting the generalizability of findings to other jurisdictions. Second, the main outcome measure was based on client-reported receipt of preventive care. The extent to which the receipt of such care in this study is either an over- or under-estimate of the care received, particularly amongst people with a mental illness is unknown [103, 104]. Direct comparison between client report outcomes and the monthly performance reports was not possible; however, the authors can confirm that the performance reports were consistent with the pattern of results reported. Third, systematic review evidence has suggested that inadequate implementation fidelity and integrity may be explanatory factors in trials that fail to show an effect [105]. In the current trial, not all intervention strategies were implemented as planned (Table 4), and there was inconsistent implementation of the intervention between the two groups. It is unknown what impact this may have had on the trial outcomes.

## Conclusions

The observed lack of an increase in preventive care provision for almost all outcome measures suggests that an intervention better tailored to the circumstances of community mental health services may be required, or one that is more intensive or includes a longer intervention period, or that an alternative model of delivering preventive care to clients of community mental health services may be required. Regardless of the specific approach, the need for a greater understanding of the barriers and facilitators to the provision of preventive care in community mental health services is indicated.
